# A rare lethal case of severe acute necrotizing pancreatitis due to a parathyroid adenoma in a third-trimester pregnant woman

**DOI:** 10.1186/s12902-019-0409-9

**Published:** 2019-07-29

**Authors:** Jun Yang, Meng-jie Dong, Feng Chen

**Affiliations:** 1 Department of Nuclear Medicine, the First Affiliated Hospital, College of Medicine, Zhejiang University, Hangzhou, 310003 People’s Republic of China; 2 Department of Radiology, the First Affiliated Hospital, College of Medicine, Zhejiang University, Hangzhou, 310003 People’s Republic of China

**Keywords:** Pancreatitis, Parathyroid adenoma, Hyperparathyroidism, Pregnant

## Abstract

**Background:**

Primary hyperparathyroidism (PHPT), which is mostly caused by a parathyroid adenoma, is fairly common in postmenopausal women but is relatively rare in pregnant women. PHPT-induced pancreatitis during pregnancy is associated with significant maternal and foetal morbidity and mortality. Diagnosis is challenging because of non-specific symptoms and changes in maternal calcium homeostasis. Information about the optimal treatment strategy for the prevention of catastrophic consequences to the mother and foetus is limited. Here, we describe a rare lethal case of severe acute necrotizing pancreatitis due to a parathyroid adenoma in a woman in her third trimester of pregnancy.

**Case presentation:**

A previously healthy 24-year-old Chinese woman at 37 weeks of gestation presented with persisting epigastric pain, nausea and bilious vomiting for 1 day. PHPT-induced acute necrotizing pancreatitis was diagnosed on the basis of her serum calcium, parathyroid levels and imaging results. A caesarean section and parathyroidectomy were performed at 1 day and 11 days after admission, respectively. Histological examination confirmed a right inferior parathyroid adenoma with a size of 2.0 × 1.5 cm. Following the parathyroidectomy, the patient had eucalcaemia and presented normal parathyroid hormone (PTH) levels. Although the foetus was normal, the patient died of multiple organ failure due to severe pancreatitis.

**Conclusions:**

PHPT-induced acute necrotizing pancreatitis is a rare clinical entity and life-threatening condition to both the mother and the foetus during pregnancy. Early diagnosis can be challenging and is crucial. Appropriate treatment according to the patient’s condition may effectively reduce maternal and foetal mortality.

## Background

Primary hyperparathyroidism (PHPT), the most common cause of hypercalcaemia, predominates among postmenopausal women and has a female:male ratio of 3–4:1 [1]. PHPT is most commonly associated with a solitary parathyroid adenoma (85–90%) but may also be associated with multigland disease (10%) and parathyroid carcinoma (< 1%) [2]. PHPT is diagnosed on the basis of the persistent elevation of serum calcium levels with corresponding elevated or inappropriately normal PTH levels [3]. The manifestations of PHPT often vary due to hypercalcaemia itself and its effects on target organs. Symptoms of PHPT include polyuria, osteopenia, depression, constipation, vomiting, and even potentially life-threatening hypercalcemia and pancreatitis [1]. PHPT during pregnancy occurs rarely and often goes undiagnosed due to a lack of symptoms, non-specific presentation and gestational physiological changes [4]. Worsening hypercalcaemia may cause acute pancreatitis during pregnancy or after delivery. Few cases of pancreatitis due to PHPT during pregnancy have been reported, and those that have been reported were generally mild oedematous pancreatitis. Early diagnosis, appropriate management and individual treatment of PHPT in pregnancy is crucial, as PHPT may infer a high risk of maternal and foetal morbidity/mortality [5, 6]. We present a rare lethal case of a 24-year-old pregnant woman presenting with severe acute necrotizing pancreatitis due to parathyroid adenoma at 37 weeks gestation.

## Case presentation

A previously healthy 24-year-old Chinese woman was admitted to the emergency department at 37 weeks of gestation because of a sudden attack of persisting epigastric pain accompanied by nausea and bilious vomiting for 1 day. Past medical and routine obstetric examinations were unremarkable. She denied a history of familial endocrine tumours and there was no history of alcohol abuse or smoking. Her vital signs were stable, and a physical examination revealed rebound tenderness in the epigastric area. The uterine size was compatible with the period of gestation, and the cervix was dilated 1 cm and hard. The foetal heart rate was a reassuring 145 beats per minute.

Initial laboratory data showed the following: white blood cell count, 28.11 × 10^9^ /L (neutrophils 90%); serum amylase, 2861 U/L (normal values < 137 U/L); lipase, 10394 U/L (normal values < 100 U/L); creatinine 111 μmol/L (normal 44–80 μmol/L); calcium, 3.11 mmol/L (normal 2.08–2.60 mmol/L); ionized calcium, 1.77 mmol/L (normal 1.10–1.34 mmol/L); phosphorus, 0.91 mmol/L (normal 0.81–1.45 mmol/L); and magnesium, 1.22 mmol/L (normal 0.70–1.10 mmol/L). Her liver function and triglyceride were normal. An ultrasound examination revealed the patient had exudative pancreatitis with peripancreatic fluid as well as bilateral nephrolithiasis and biliary sludge without evidence of cholelithiasis. The patient was diagnosed with acute pancreatitis. Her pancreatitis was managed with fasting, intravenous fluids, analgesics, and empirical antibiotics; however, her clinical status and laboratory parameters did not improve. A caesarean delivery with spinal anaesthesia was performed the next day after the patient provided consent and discussed the procedure with a multidisciplinary team for high foetal risk. A healthy boy weighing 2620 g with an Apgar score of 9 and 9 at 1 and 5 min, respectively, was delivered.

**Fig. 1:**
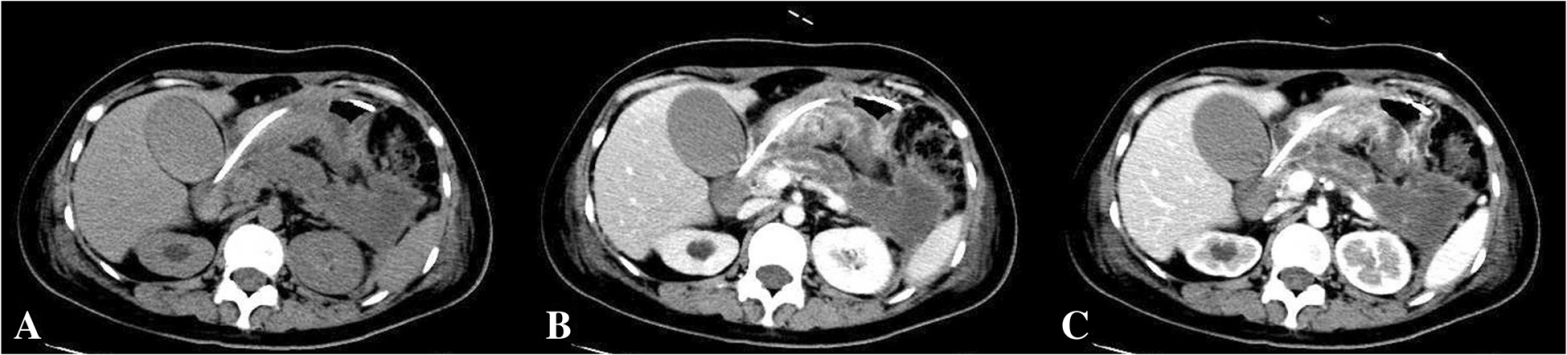
Abdominal CT scan. It showed diffuse enlarged pancreas with indistinct boundaries and surrounding exudates (**a**: non-contrast-enhanced image; **b**: arterial phase image; **c**: delayed phase image). CT = computed tomography

**Fig. 2:**
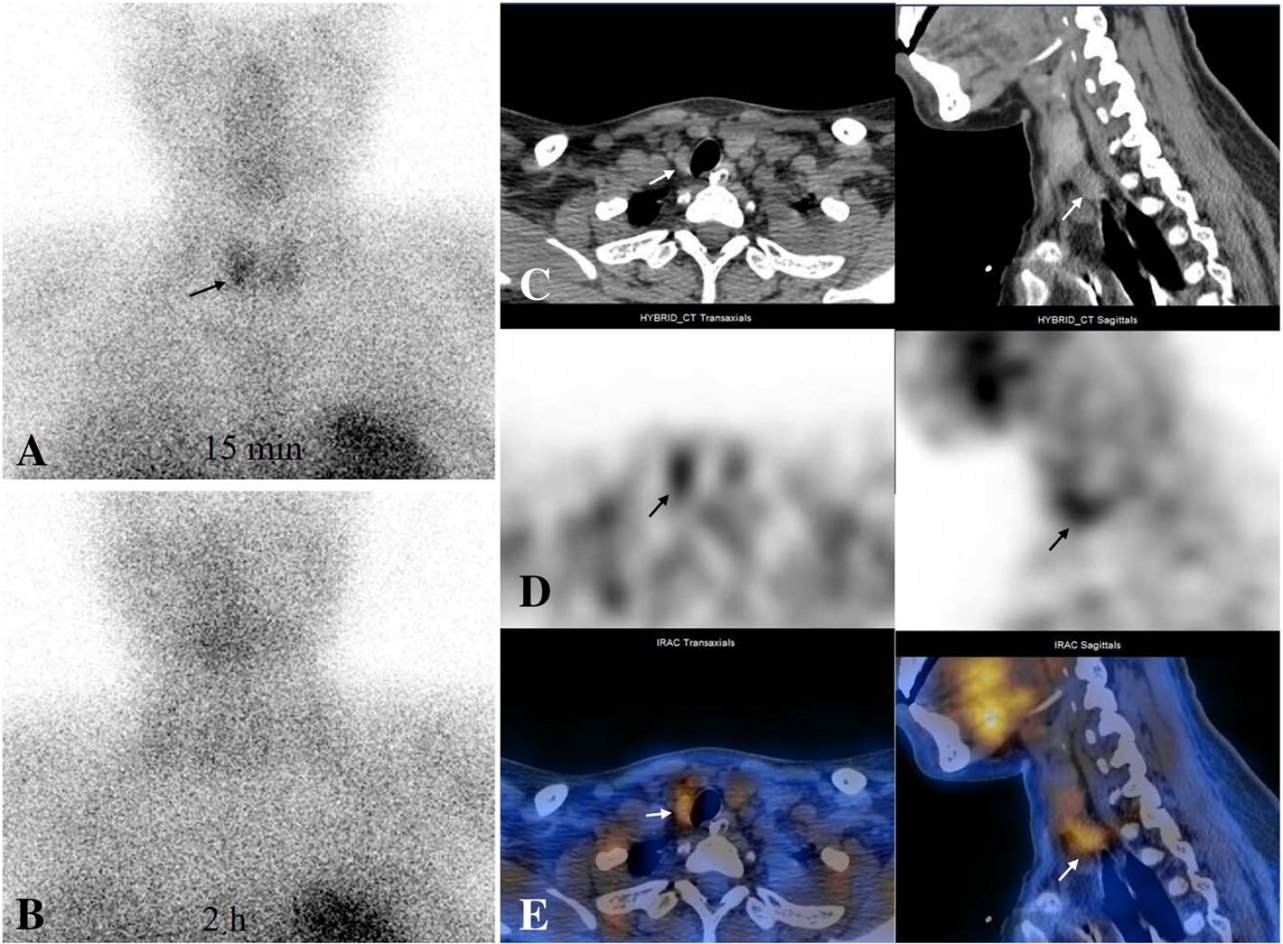
^99m^Tc-MIBI scintigraphy and SPECT/CT. ^99m^Tc-MIBI scintigraphy demonstrating increased uptake in the inferior aspect of the right thyroid lobe (arrow) on early acquisition **a**; rapid ^99m^Tc-MIBI clearance at 2 h delayed phase **b**; ^99m^Tc-MIBI SPECT/CT acquisition depicting intense radiotracer accumulation (arrow) localizing to an oval nodule, consistent with a right inferior parathyroid adenoma (**c**: axial and sagittal CT; **d**: axial and sagittal SPECT; **e**: fused axial and sagittal images). ^99m^Tc-MIBI = technetium-99 m-sestamibi; SPECT/CT = single photon emission tomography/computed tomography

**Fig. 3:**
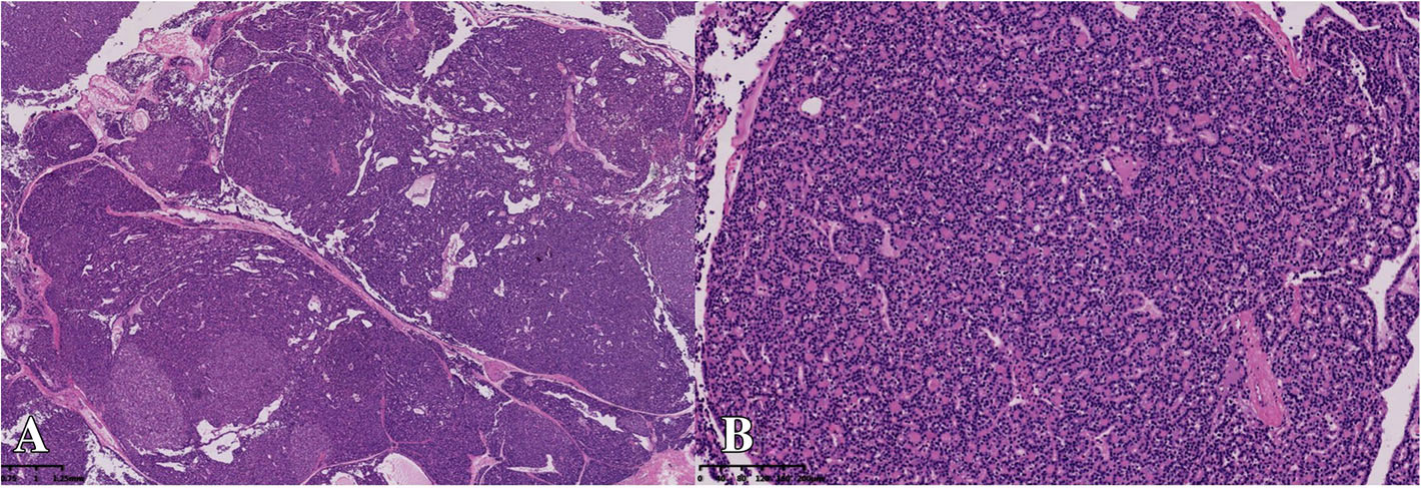
Hematoxylin and eosin staining of the parathyroid adenoma. Magnificationx20 (**a**) and × 100 (**b**). The adenoma was composed mainly of chief cells

The patient’s body temperature increased to 39.0 °C 3 days after surgery. Her symptoms of pancreatitis worsened, and an abdominal enhanced computed tomography (CT) scan revealed severe necrotizing pancreatitis (Fig. 1a, b and c). The aetiology of pancreatitis showed that her initially ionized calcium levels were increasing, with a value of 1.8 mmol/L (normal 1.10–1.34 mmol/L) as well as low serum phosphorus levels. Further laboratory evaluation showed an increased PTH level (500 pg/mL; normal levels, 12–65 pg/mL) with a normal 25-hydroxyvitamin D (34 nmol/L) plasma level (normal levels, 12.3–107 nmol/L). Detailed relevant laboratory tests are shown in Table 1. The diagnosis of PHPT was confirmed. Technetium-99 m-sestamibi (^99m^Tc-MIBI) scintigraphy revealed an abnormal accumulation in the right inferior parathyroid region at 15 min, and rapid ^99m^Tc-MIBI clearance was observed in delayed 2-h images (Fig. 2a and b). The accurate localization of a parathyroid adenoma with a size of 2 cm was achieved with single photon emission computed tomography (SPECT)/CT (Fig. 2c, d and e). A parathyroidectomy was performed 10 days after childbirth under general anaesthesia. A single right inferior parathyroid adenoma 2.0 × 1.5 cm in size was completely removed. Histological examination revealed the chief-cell type of the parathyroid adenoma (Fig. 3a and b). Her calcium and PTH levels returned to normal 24 h after surgery, and hypocalcaemia occurred 3 days after surgery. However, the clinical situation of pancreatitis aggravated rapidly, and ultrasound revealed massive necrotizing tissue around the pancreas. Despite a necrosectomy with evacuation of the tissue debris and repeated percutaneous drainage, the patient developed multiple organ failure and died 72 days after childbirth. The baby boy grew uneventfully during follow-up.

**Table 1:** Results of initial laboratory values

Parameters	Parameter value	Reference range
White blood cell count (10^9^/L)	**28.11**	4.0–10.0
Neutrophil (10^9^/L)	**25.30**	2.0–7.0
Hemoglobin (g/L)	130	113–151
Platelets (10^9^/L)	264	101–320
Fasting glucose (mmol/L)	4.5	3.9–6.1
Albumin (g/L)	37.7	35–55
Alanine aminotransferase (U/L)	11	3–35
Aspartate aminotransferase (U/L)	15	8–40
Total bilirubin (μmol/L)	6.9	0–21
Alkaline phosphates (U/L)	51	40–150
Lipase (U/L)	**10394**	1–100
Serum amylase (U/L)	**2861**	0–137
Creatinine (μmol/L)	**111**	44–80
Total cholesterol (mmol/L)	4.56	3.14–5.86
Triglycerides (mmol/L)	1.38	0.30–1.70
Potassium (mmol/L)	4.43	3.50–5.20
Sodium (mmol/L)	137.2	136–145
Calcium (mmol/L)	**3.11**	2.08–2.60
Ionized calcium (mmol/L)	**1.77**	1.10–1.34
Magnesium (mmol/L)	1.22	0.70–1.10
Phosphorus (mmol/L)	**0.91**	0.81–1.45
Thyroid stimulating hormone (mIU/L)	1.273	0.38–4.34
Intact parathyroid hormone (pg/mL)	**500**	12.0–65.0
25-hydroxyvitamin D (nmol/L)	34	12.3–107

Bolded values are out of the reference range

## Discussion and conclusions

PHPT refers to an intrinsic parathyroid gland abnormality that produces the excessive secretion of parathyroid hormone and causes hypercalcaemia [1]. It is an uncommon clinical entity in women of childbearing age. The prevalence of PHPT during pregnancy is unknown and may occur in less than 1% of cases [7]. In fact, many authors believe that the true prevalence in pregnancy is underestimated because of physiological pregnancy changes and mild non-specific symptoms [8, 9]. Early diagnosis and appropriate intervention for the mother and foetus are crucial to prevent problems, such as nephrolithiasis, bone disease, preeclampsia, spontaneous abortion, intrauterine demise, and lethal pancreatitis or severe hypercalcaemia [9]. Potentially life-threatening acute pancreatitis is also rare during pregnancy, especially acute pancreatitis due to PHPT [10]. Clinical knowledge about pancreatitis due to PHPT is limited to isolated case reports, such as the case report presented here [11–17].

Diagnosis of PHPT during pregnancy is always a challenge owing to the uncommonness of this metabolic disorder, non-specific symptoms and lack of routine serum calcium tests. Additionally, calcium homeostasis during pregnancy changes as calcium is provided for the developing foetus. Patients with PHPT usually present only mildly elevated serum calcium levels and are otherwise asymptomatic [9]. Several reasons, including intravascular fluid expansion, gestational hypoalbuminemia, increased glomerular filtration, transplacental calcium transfer and increased levels of oestrogen, may contribute to the decreased calcium levels and masking of PHPT [18]. When the protective effects of pregnancy are removed, maternal PHPT may worsen. Abruptly increased maternal hypercalcaemia may induce pancreatitis and hypercalcaemic crisis, especially during the third trimester or in the post-partum period. It had been reported as well [12, 13, 18, 19]. PHPT-induced pancreatitis during pregnancy has been explained as follows: a high concentration of calcium in the pancreatic juice activates trypsinogen secretion. Calcifications in the pancreatic ducts and expanded pregnant uterus in the peritoneal cavity block secretions, and increased levels of parathyroid hormone directly injure the pancreas [20, 21]. The most common symptoms in patients with pancreatitis due to PHPT are abdominal pain and vomiting, which were also observed in our patient. Although hypocalcaemia may present in patients with severe acute pancreatitis, hypercalcaemia is still an important clue for the diagnosis of PHPT. When we excluded the other aetiologies of acute pancreatitis, the patient was diagnosed with PHPT-induced acute necrotizing pancreatitis. Only a few cases have been reported [14, 21].

There is no consensus on the treatment of PHPT-induced pancreatitis during pregnancy. Most physicians agree that treatment should be individualized and take into account the patient’s symptoms, the severity of hypercalcaemia, the gestational age and the risk-benefit balance of each treatment [4]. Conservative treatments are recommended in asymptomatic patients with mild hypercalcaemia (< 2.75 mmol/L), and mothers and foetuses usually have good outcomes [4, 22]. Parathyroidectomy is the only curative treatment and is recommended when calcium levels are above 2.75 mmol/L. The optimal time for surgery has been considered to be the second trimester. In our patient, due to a delayed diagnosis of PHPT in the third trimester, acute necrotizing pancreatitis, persistent hypercalcaemia and a potential threat to the foetus, a caesarean section and parathyroidectomy were performed immediately at 37 weeks of gestation. Following the parathyroidectomy, the foetus was normal, and the patient was eucalcaemic and had normal PTH levels; however, the patient died of multiple organ failure due to severe pancreatitis.

In conclusion, although PHPT-induced acute pancreatitis is very rare and most patients have mild hypercalcaemia or are asymptomatic, serious complications may have catastrophic consequences for both the mother and the foetus. Diagnosis and treatment can be challenging, and severe complications are life threatening. Our case indicates that early diagnosis and appropriate management may reduce maternal and foetal mortality.

## Data Availability

The datasets supporting the conclusions of this article is included within the article and in figures.

## Abbreviations

^99m^Tc-MIBI: Technetium-99 m-sestamibi
CT: Computed tomography
PHPT: Primary hyperparathyroidism
SPECT/CT: Single photon emission tomography/computed tomography
